# Metabolomics-based investigation of SARS-CoV-2 vaccination (Sinovac) reveals an immune-dependent metabolite biomarker

**DOI:** 10.3389/fimmu.2022.954801

**Published:** 2022-09-28

**Authors:** Maozhang He, Yixuan Huang, Yun Wang, Jiling Liu, Maozhen Han, Yixuan Xiao, Na Zhang, Hongya Gui, Huan Qiu, Liqing Cao, Weihua Jia, Shenghai Huang

**Affiliations:** ^1^ Department of Microbiology, The Key Laboratory of Microbiology and Parasitology of Anhui Province, The Key Laboratory of Zoonoses of High Institutions in Anhui, School of Basic Medical Sciences, Anhui Medical University, Hefei, China; ^2^ Department of Clinical Medicine, The First School of Clinical Medicine, Anhui Medical University, Hefei, China; ^3^ Department of Nosocomial Infection Control, Anhui No.2 Provincial People’s Hospital, Hefei, China; ^4^ School of Life Sciences, Anhui Medical University, Hefei, China; ^5^ School of Nursing, Anhui Medical University, Hefei, China

**Keywords:** COVID-19, SARS-CoV-2 vaccination, serum, antibodies and cytokines, metabolomic analysis

## Abstract

SARS-CoV-2 and its mutant strains continue to rapidly spread with high infection and fatality. Large-scale SARS-CoV-2 vaccination provides an important guarantee for effective resistance to existing or mutated SARS-CoV-2 virus infection. However, whether the host metabolite levels respond to SARS-CoV-2 vaccine-influenced host immunity remains unclear. To help delineate the serum metabolome profile of SARS-CoV-2 vaccinated volunteers and determine that the metabolites tightly respond to host immune antibodies and cytokines, in this study, a total of 59 sera samples were collected from 30 individuals before SARS-CoV-2 vaccination and from 29 COVID-19 vaccines 2 weeks after the two-dose vaccination. Next, untargeted metabolomics was performed and a distinct metabolic composition was revealed between the pre-vaccination (VB) group and two-dose vaccination (SV) group by partial least squares-discriminant and principal component analyses. Based on the criteria: FDR < 0.05, absolute log2 fold change greater than 0.25, and VIP >1, we found that L-glutamic acid, gamma-aminobutyric acid (GABA), succinic acid, and taurine showed increasing trends from SV to VB. Furthermore, SV-associated metabolites were mainly annotated to butanoate metabolism and glutamate metabolism pathways. Moreover, two metabolite biomarkers classified SV from VB individuals with an area under the curve (AUC) of 0.96. Correlation analysis identified a positive association between four metabolites enriched in glutamate metabolism and serum antibodies in relation to IgG, IgM, and IgA. These results suggest that the contents of gamma-aminobutyric acid and indole in serum could be applied as biomarkers in distinguishing vaccinated volunteers from the unvaccinated. What’s more, metabolites such as GABA and taurine may serve as a metabolic target for adjuvant vaccines to boost the ability of the individuals to improve immunity.

## Introduction

As of now, SARS-CoV-2 is still ravaging the world. Vaccination is treated as an integral part of the long-term management of the COVID-19 epidemic (1, 2), which elicits protective neutralizing antibodies against SARS-CoV-2 and offers hope for containing the COVID-19 pandemic. As of 26 April 2022, more than 11.32 billion doses of the vaccine have been administered worldwide (3), which have been reported with substantial efficacy against SARS-CoV-2 and its variants (4–6). Recent newly emerging variants, combined with the steady decline in antibody levels among vaccinated individuals, have implied a resurgence of the epidemic over time (7–9). The Sinovac-CoronaVac COVID-19 vaccine is licensed as a vaccine for the prevention of COVID-19 and against emerging SARS-CoV-2 coronavirus variants in China. Despite a large-scale population-based cohort safety evaluation of SARS-CoV-2 vaccines, these prior studies have disregarded the underlying function of biologically active metabolic small molecules between different immune cells in the organism in triggering the immune response associated with vaccination. The high throughput-based omics techniques to study systemic vaccinology at the cellular and molecular levels allow for a comprehensive overview of the interrelationship between vaccination and vaccine-induced immune responses.

There have been a handful of proteomic- and/or metabolomics-based studies since the COVID-19 outbreak that revealed a previously unrecognized metabolic profile associated with SARS-CoV-2 infection and disease severity (10–12). In general, liquid chromatography–mass spectrometry (LC/MS)-based metabolomics studies identified the dysregulation of metabolites such as butyric acid, 2-hydroxybutyric acid, l-phenylalanine, and kynurenine metabolites in the serum of COVID-19 patients (13, 14). However, there have been few prospective studies involving metabolomics-based studies of SARS-CoV-2 vaccination-induced immune responses in the human population. To date, there have been only two studies based on metabolomics to investigate the alteration of serum metabolites in the host metabolism after the inoculation of the COVID-19 vaccine and the network interplay with the immune response, and they mainly identified metabolites in the processes of tricarboxylic acid cycle, amino acid metabolism, and lipid metabolism associated with the immune response (15, 16). Nevertheless, there is a paucity of studies on the interaction between circulating metabolites and vaccine-induced antibody responses after COVID-19 vaccination.

Metabolism is the basic characteristic and main activity of life. LC/MS-based metabolomics is widely used in the mechanistic study and diagnosis of various diseases, such as cancer, diabetes, and cardiovascular or pulmonary disorders upon infection of a virus (17, 18). A growing number of studies have underscored the impact that the metabolic state of an organism can exert on the shaping of the body’s immune system (19–21). For example, a preceding study revealed a confidential link between metabolic phenotype and vaccine immunity in healthy individuals (22). In addition, findings from clinical or animal studies suggest that the metabolites derived from gut microbiota are critical in regulating the immune responses to vaccination. Various gut bacterial metabolites have been reported to be implicated in modulating the response of vaccine immunity, such as short-chain fatty acids (SCFAs), bile acids, and aromatic amino acids (AAAs). Short-chain fatty acids are the best documented to have many beneficial benefits to the individual, ranging from but not limited to the regulation of glucolipid metabolism, inflammation, immunity, and tumors (23). A previous work has noted that SCFAs could potentiate the expression of genes involved in plasma cell differentiation and class transformation, which provides the energy requirements for the antibody response in the non-pathogenic infection state and in the presence of pathogenic infection (24).

To further elucidate and provide a comprehensive insight into whether COVID-19 vaccines influence the response of host serum metabolites and the interplay between metabolites and vaccine-stimulated antibodies and cytokines, in this study, a metabolomic analysis has been applied to screen for metabolic variations and to provide a comprehensive view of endogenous metabolites in relation to the vaccination. We conducted a prospective observational study of undergraduates who received the inactivated SARS-CoV-2 vaccine (CoronaVac; Sinovac) to examine the serum metabolic determinants of immunization responses.

## Volunteers and methods

### Study design and participants

The study recruited a total of 30 healthy undergraduates as participants from Anhui Medical University. We collected the sera of those volunteers before vaccination in 9 May 2021 and after receiving two doses of the inactivated COVID-19 vaccine (CoronaVac, Sinovac Life Sciences, Beijing, China; N = 30) in 21 June 2021 at the First Affiliated Hospital of Anhui Medical University. Eligible participants recruited from Anhui Medical University were aged 18–20 years old, had a well-regulated lifestyle, had eaten in the university canteen for the past 2 months, and had no history of SARS-CoV-2 infection upon inoculating the CoronaVac vaccine. The exclusion criteria were volunteers who had a history of allergies, had a history of medication, and received antibiotic drugs in the last 2 months; these were determined by means of a questionnaire. All participants provided written informed consent and completed two doses of the vaccine. All individuals enrolled in this study were asked to sign an informed consent before the investigation, following the principle of informed consent and strict confidentiality, which has been approved by the ethics committee of Anhui Medical University Biomedical Ethics Committee (Approval No. 2021H021). Each individual or his/her legal guardian provided the signed informed consent before enrolment. All volunteer data sets were anonymized.

### Measurement of indicators using routine blood tests

Various indicators obtained from routine blood tests, such as eosinophils, neutrophils, and hemoglobin, were measured *via* using routine blood tests conducted at Hefei City Maternal and Child Health and Family Planning Service Center (Anhui Province). Specifically, a 2-ml blood sample was collected in an EDTA-K2 anticoagulant tube. Immediately after collection, the tube was mixed by being gently reversed several times to ensure adequate anticoagulation of the blood specimen. The blood samples were then sent to Hefei City Maternal and Child Health and Family Planning Service Center, and 18 indicators, such as white blood cell (WBC), neutrophil (Neu), monocytes (Mon), red blood cell (RBC), and hematocrit value (HCT), obtained from routine blood tests, were directly measured using a three-classification blood cell analyzer (BC-5390 CRP, Marry). The results from the routine blood tests of all blood samples are summarized in **Table S1**.

### Serum sample collection and pretreatment

The elbow vein blood (5 ml) was collected from volunteers before vaccination and after receiving two doses of the vaccine at 6 a.m. to 8 a.m. in a fasting state with vacuum negative-pressure blood collection vessels, and was immediately centrifuged for 15 min (3,500 rpm, 4°C). Each aliquot (0.5 ml) of the serum samples was prepared and stored at –80°C until use (25). Then, all samples were thawed on ice before the metabolite profiling (26). Untargeted metabolomics of sera samples was performed as previously described (27), with slight modification. In brief, 80 µl of sample was mixed with 320 μl of acetonitrile by vortexing for 60 s. Then, the sample was centrifuged at 18,920×*g* for 10 min (4°C) to precipitate the protein. Two 150-μl aliquots of the supernatant were transferred and lyophilized for analysis in positive and negative electrospray ionization (ESI^+^ and ESI^−^) mode, respectively. Fifty microliters of a 25% (by volume) acetonitrile aqueous solution was used to reconstitute the sample before the LC-MS analysis. Quality control (QC) samples were prepared by pooling the same volume of each sample to evaluate the reproducibility of the analysis. The pretreatment of the QC samples paralleled and was the same as that of the study samples. The QC samples were evenly inserted in each set of the analysis running sequence to monitor the stability of the large-scale analysis (28).

### Untargeted LC-MS/MS analysis

The metabolite profiles of serum were detected by using an LC-MS/MS system consisting of an ultra-high-performance liquid chromatography (UPLC) system (Vanquish, Thermo Fisher Scientific, Waltham, MA, USA) with a UPLC BEH Amide column (2.1 mm × 100 mm, 1.7 μm) coupled to a Q Exactive (QE) HFX mass spectrometer (Orbitrap MS, Thermo Fisher Scientific, Waltham, MA, USA). The UPLC system was used for the separation of biochemicals *via* the UPLC BEH Amide column. The mobile phase consisted of 25 mmol/L ammonium acetate (Sigma-Aldrich, Darmstadt, Germany) and 25 mmol/L ammonia hydroxide (Fisher Chemical, Waltham, MA, USA) in water (pH = 9.75) (A phase) and acetonitrile (B phase). The autosampler temperature was 4°C, and the injection volume was 3 μl. The QE HFX mass spectrometer was applied to obtain MS/MS spectra in information-dependent acquisition (IDA) mode under the control of the acquisition software (Xcalibur, Thermo Fisher Scientific, Waltham, MA, USA). In this mode, the acquisition software continuously evaluates the full-scan MS spectrum. The electrospray ion (ESI) conditions were as follows: sheath gas flow rate of 30 Arb, auxiliary gas flow rate of 25 Arb, capillary temperature of 350°C, full MS resolution of 60,000, MS/MS resolution of 7500, collision energy of 10/30/60 in negative ion mode (NCE), and spray voltage of 3.6 kV (positive) or −3.2 kV (negative).

### Serum metabolomic analysis

The original LC-MS/MS data were converted to the mzXML format using ProteoWizard and processed with an in-house program, which was developed using R and based on XCMS, for peak detection, extraction, alignment, and integration. Subsequently, for further multivariate analysis, the raw data were pretreated. Pretreatment included de-noising based on the relative standard deviation (RSD), filling the missing data *via* half of the minimum value, and the batch normalization with peak area was used to calibrate the metabolomics data. The final dataset contained the information of peak number, sample name, and normalized peak area and was imported to the SIMCA16.0.2 software package (Sartorius Stedim Data Analytics AB, Umea, Västerbotten, Sweden). Firstly, we performed a principal component analysis (PCA), an unsupervised analysis, to reduce the dimensions of the data. The 95% confidence interval (95% CI) in the PCA score plot was used as the threshold to identify potential outliers in the dataset. Secondly, in order to visualize the group separation and find significantly changed metabolites, we conducted the supervised orthogonal projections to latent structure discriminate analysis (OPLS-DA) and acquired the value of variable importance in projection (VIP). A sevenfold cross-validation test was performed to evaluate the goodness of fit of the OPLS-DA model using the values of R^2^Y and Q^2^. R^2^Y indicates how well the variation of a variable is explained, and Q^2^ indicates how well a variable can be predicted. A 200-time permutation test was then conducted to assess the robustness of the model. The precursor molecule passed the combined criteria: 1) absolute log2 fold change (|log2FC|) greater than 0.25, 2) VIP >1, and 3) adjusted p-value <0.05 (nonparametric *t*-test) were considered significantly changed between the SV and VB groups. Furthermore, after scanning for the differential metabolite features, the fragment information obtained from the MS/MS model was then further matched with the annotations in HMDB, Metlin, massbank, LipidMaps, mzclound, and in-house standards database (PPM <10) to obtain accurate metabolite information. Finally, commercial databases, including Kyoto Encyclopedia of Genes and Genomes (KEGG) (http://www.genome.jp/kegg/ (accessed on 15 June 2021) and MetaboAnalyst (http://www.metaboanalyst.ca/ (accessed on 15 June 2021), were used for pathway enrichment analysis.

### Measurement of cytokines and SARS-CoV-2 antibodies in serum by ELISA

To monitor the response of the host’s body based on cytokines and SARS-CoV-2 antibodies, the levels of inflammatory factors, including IL-2, IL-4, IFN-γ, and SARS-CoV-2 antibodies, including anti-(N+S) IgA, anti-(N+S) IgG, and anti-(N+S) IgM, were measured by ELISA. Specifically, the levels of cytokines and antibodies in whole-blood serum obtained from all of the blood samples (n = 30 and 29 at each sampling point) were measured. We selected IL-2 and IFN-γ as representatives of Th1 cells and IL-4 as the representative of Th2 cells. IL-2, IL-4, IFN-γ, anti-(N+S) IgA, anti-(N+S) IgG, and anti-(N+S) IgM were measured using a high-sensitivity enzyme-linked assay quantitative kit (the kits for cytokines were procured from Bio-Techne USA Co., Ltd., Minnesota, USA, and the kits for antibodies were obtained from Wuhan Fine Biotechnology Co., Ltd., Wuhan, China) in accordance with the manufacturer’s instructions. Specifically, taking the detection of SARS-CoV-2 antibodies as an example, the kit can detect the concentration of SARS-CoV-2 antibodies based on indirect enzyme-linked immunosorbent assay technology. Ninety-six-well plates were percolated with recombinant 2019-nCoV nucleocapsid and spike protein (antigen), and an HRP-conjugated antibody was used as the detection antibody. Subsequently, the standards, test samples, and HRP-conjugated detection antibody were added to the wells, and the plates were washed with wash buffer. 3,3′,5,5′-Tetramethylbenzidine (TMB) substrates were used to visualize the HRP enzymatic reaction, which is catalyzed by HRP to produce a blue product that changes to yellow after the addition of an acidic stop solution. The density of the yellow color is proportional to the target amount of the sample captured in the plate. The O.D. absorbance at 450 nm was read using a microplate reader, and the concentration of the target was then calculated.

### Statistical analysis

Differential serum metabolites were identified using the Wilcoxon test and the p-value was adjusted for multiple testing *via* the false-discovery rate (FDR) using the Benjamini-Hochberg method. An R package Randomforest (version 4.6.14) was used to select important variables in the serum data and build a machine learning model to distinguish whether participants had received the SARS-CoV-2 vaccine. For feature selection, 100 endogenous serum metabolites screened from the 156 distinct metabolites between the VB and SV groups were selected as input features. Finally, the 50 metabolites, whose mean decrease accuracy ranked in the top 50, were determined by 10-fold cross validation using the *rfcv* function. After selecting 50 metabolites, we adopt binary logistic regression to build the model based on the potential biomarkers. A receiver-operating characteristic curve was used to evaluate the results of the regression analysis. For network analysis, we used Cytoscape (version 3.9.1) (29) to visualize the correlation relationships calculated by the SparCC algorithm (30) among metabolites, while the biologically important subnetworks were identified using the Molecular Complex Detection (MCODE) plugin in the Cytoscape software (31). In addition, a correlation analysis between metabolites and clinical parameters was also performed using Spearman rank correlation analysis.

Apart from data related to metabolomics, other experimental data were analyzed using the GraphPad Prism 9 software (GraphPad Software, La Jolla, CA, USA). The data with a normal distribution were represented as mean ± standard deviation (mean ± SD), and differences between groups were compared using *t*-test. All statistical tests were two-tailed, and the differences reached the statistical significance which was set at p < 0.05.

## Results

### SARS-Cov-2 vaccine-induced vigorous antibody responses

**Figure 1:**
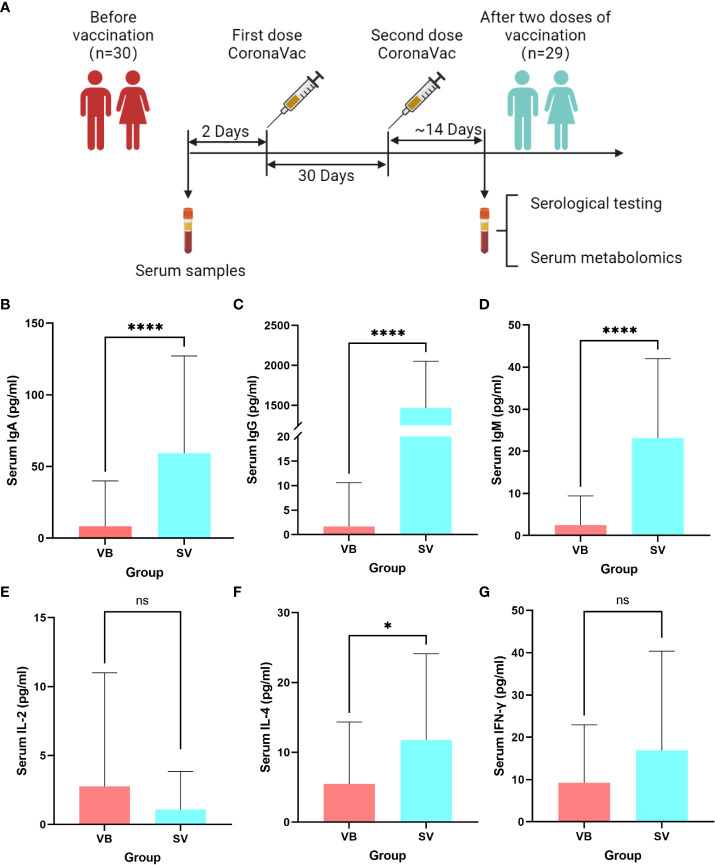
Untargeted metabolomic profiling of sera samples collected from volunteers before SARS-CoV-2 vaccination, and 2 weeks after two-dose vaccination. **(A)** Schematic diagram of the study design. **(B–G)** Antibodies and cytokines characterized in serum and compared between the VB and SV groups. ns indicates not significant, single asterisk indicates p < 0.05, double asterisks indicate p < 0.01, triple asterisks indicate p < 0.001, quadruple asterisks indicate p < 0.0001.

We recruited a cohort of college students, and finally, 29 individuals comprising 15 men and 14 women were included in this study for none antibiotic use within 2 months. A serum sample was obtained from each volunteer before vaccination (VB) and 2 weeks after the second vaccination (SV). We analyzed 24 clinical serum measurements of the nonvaccinated and vaccinated subjects (**Figure 1A**, **Table 1**), involving six immunoglobulins of IgA, IgG, and IgM; Th1 cytokines of IL-2 and IFN-γ; Th2 cytokines of IL-4; and 18 blood routine indicators. Compared to non-vaccinated individuals, volunteers who received two doses of the SARS-CoV-2 vaccine showed significant upregulation of IgA and IgM, as well as an increase in IgG (**Figures 1B–D**). However, IL-4 was only found to be significantly different between the VB and SV groups as with cytokines (**Figures 1E–G**). Furthermore, we found that the levels of five routine blood indicators (RBIs), mean corpuscular volume (MCV), red blood cell distribution width-standard deviation (RDW-SD), mean platelet volume (MPV), platelet-large cell count (P-LCC), and platelet-large platelet ratio (P-LCR), showed a significant decreasing trend in the SV group compared to participants in VB group. However, the levels of other RBIs, such as white blood cells (WBC), red blood cells (RBC), neutrophil (Neu), and eosinophil (Eos) did not reach statistical significance (**Figure S1**).

**Table 1:** Characteristics of the serum levels of cytokines and antibodies in the VB and SV groups.

Group	IFN-γ	IL-2	IL-4	IgA	IgG	IgM
	Mean ± SD						
*VB	9.25 ± 13.70	2.77 ± 8.24	5.49 ± 8.87	8.57 ± 32.25	1.72 ± 9.10	2.54 ± 7.03
*SV	16.89 ± 23.46	1.08 ± 2.77	11.77 ± 12.37	59.26 ± 67.92	1466.24 ± 585.80	23.13 ± 18.87

*VB: before vaccination; SV: two-dose vaccination.

### Metabolomic profiling of sera from volunteers who received COVID-19 vaccines

We performed untargeted metabolomics based on ultra-performance liquid chromatography/tandem mass spectrometry (UPLC-MS/MS) to analyze the sera samples. The typically extracted ion chromatograms (base peak chromatogram) from two ESI modes are displayed in **Figure S2**. The coefficients of variation of the peak distribution in the QC samples showed that this analysis was stable and repeatable (**Figure S3**). Additionally, in the principal component analysis score plots of both ESI^+^ and ESI^-^ (**Figures S4A, B**), the QC samples clustered tightly together, which further confirmed the reliability of the present study. Furthermore, the discrimination trends among samples between the VB and SV groups revealed obvious separation (**Figures S4C, D**). After peak alignment, peak picking, and deconvolution, we detected a total of 17,072 and 13,286 precursor molecules in positive and negative ion modes, respectively. We further excluded the metabolic features of which the percentage of relative standard deviation (RSD%) was more than 30% in QC samples, and each peak presented (non-zero value) in more than 50% of total samples was included in the data analysis. Ultimately, 15,485 and 10,411 putative metabolic features in the positive and negative ion modes were obtained and merged together for further analysis, respectively. Altogether, 2376 metabolites were identified and quantified based on searches of online databases (HMDB and Metlin) or confirmed with authentic standards from PANOMIX. These variables were used for the subsequent multivariate and univariate analyses. The details of the metabolites are shown in **Table S2**. We further checked the influence of sex to the metabolomic profiling. As shown in the UMAP plots (**Figure S5**), we did not observe sex-biased influence on the metabolomic data.

### Apparent alteration of serum metabolites associated with SARS-Cov-2 vaccination

**Figure 2:**
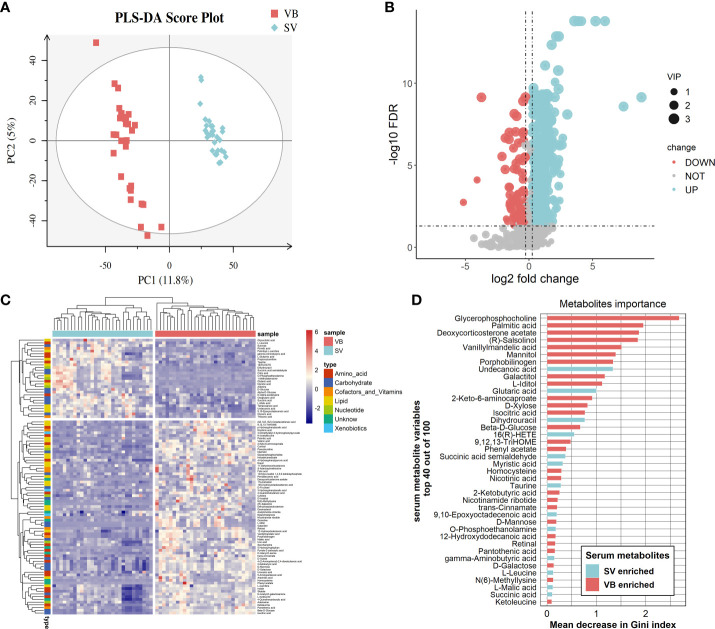
Alteration of main metabolites in sera samples from the vaccinated individuals. **(A)** Partial least squares discriminant analysis score plot based on the VB and SV groups. **(B)** Volcano plots highlight the serum metabolites that were increased (red) in the SV compared to the VB group, with FDR < 0.05, log2 fold change (FC) >0.25 or <−0.25. **(C)** Heatmap clustering of distinct serum metabolites from the comparison between the VB and SV groups. **(D)** Variable importance plot of the top 40 serum metabolites (y-axis) ranked by the contribution to mean decrease accuracy of the Gini coefficient (x-axis) in the random forest model for discerning group difference.

First, the partial least squares discriminant analysis score plot (**Figure 2A**) revealed clear separations between the VB and SV groups without overfitting (**Figure S6**). To reveal the serum metabolic characteristics in volunteers who received two doses of the vaccine and identify and confirm high-confidence metabolites associated with vaccination, we distinguished metabolites based on the criteria of a log_2_ fold change (FC) ≥0.25 or ≤-0.25 and adjusted p value (FDR) <0.05, respectively. Thus, metabolic characteristics with significant differences were extracted and visualized by volcano plots (**Figures 2B**). In addition, a metabolite with a VIP value greater than 1.0 was selected as a significantly different metabolic feature for further analysis. Finally, 100 out of 156 differential metabolites that were endogenous (56 non-endogenous were discarded) were reserved, containing 60 and 40 metabolites from the ESI^+^ and ESI^-^ models, respectively. Hierarchical clustering analysis and heatmap also revealed differentially expressed metabolites between VB and SV. These include the enrichment of L-glutamic acid, L-leucine, GABA, taurine, and succinic acid. Interestingly, L-leucine, L-glutamic acid, succinic acid, and citric acid were common differentially expressed metabolites between SARS-CoV-2 infection and immunization, but with the opposite expression (32). Among them, we observed a significant correlation between taurine and antibody levels (**Figure S7**). In contrast, indole, phenyl acetate, and L-carnitine were depleted in SV individuals compared with the VB group (**Figure 2C**). Next, we focused on individual serum metabolites for in-depth analyses to delineate potential correlations with vaccination. Random forest (Rf) analyses ranked all altered 100 serum metabolites by contribution to the group separation; the top 50 differentiating metabolites are shown (**Figure 2D**, **Figure S7D**). Notably, we noted several metabolites that were statistically different between the VB and SV groups, which were also confirmed as high-impact metabolites with huge importance in Rf analysis. For example, phenyl acetate which ranked sixteenth and L-iditol which ranked eighteenth had 8.6- and 44.1-fold elevations in the VB group.

### Network analysis and metabolic pathway analysis

**Figure 3:**
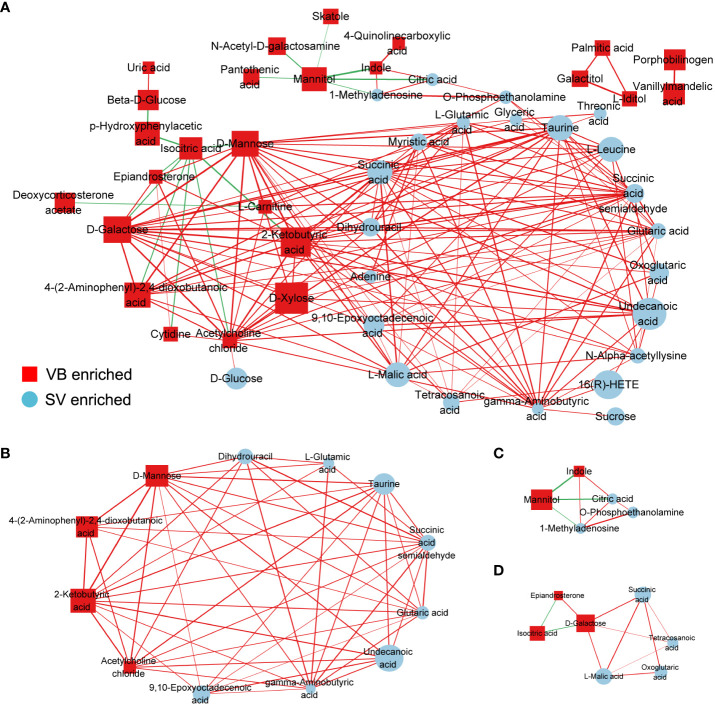
Correlation coefficients-based network constructed by distinct metabolites between the VB and SV groups. **(A)** The nodes were colored and shaped by different groups (ellipses represent SV group, and rectangle stands for the VB group). The color of the edge is set to red and green, which represent positive and negative correlation, respectively. The width of the edge indicates the magnitude of the correlation coefficient. **(B–D)** The three subnetworks were identified by the MCODE plugin in the Cytoscape software.

**Figure 4:**
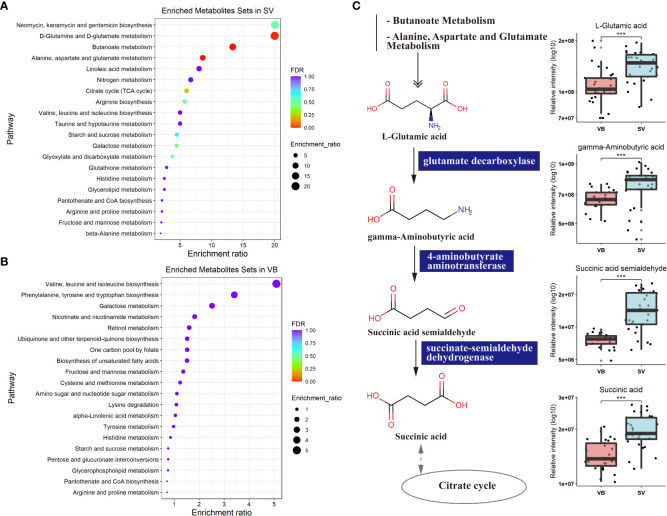
Metabolic KEGG enrichment analysis based on the differential metabolites between groups. **(A)** KEGG pathway analysis of differentiating metabolites enriched in the SV group. The color of the bubbles represents the value of adjusted p value, and the size of bubbles represents the number of counts (sorted by enrichment ratio). **(B)** KEGG pathway analysis of distinguishing metabolites increase unique to the VB group. **(C)** Schematic diagram of four SV-enriched metabolites participating in the butanoate metabolism and the alanine, aspartate, and glutamate metabolism KEGG pathways. Triple asterisks indicate p < 0.001.

To further explore the potential regulatory relationships using the vaccination-related differential metabolites, a correlation network was constructed based on the correlation relationship that the correlation coefficients and p-value should be reached based on the criteria |r| > 0.3 and p < 0.05 (**Table S3**). On the whole, the interaction network comprised 49 nodes and 117 interactions. We observed that the metabolites enriched in SV group were almost showing positively correlated with each other, such as taurine, succinic acid, glutaric acid, and GABA. However, we also detected that the metabolites depleted in the SV group exhibited a positive association with those that increased in the SV group, for instance, D-mannose, D-galactose, and 2-ketobutyric acid (**Figure 3A**). In addition, molecular complex detection (MCODE) analysis identified three key subnetworks (module 1, module 2, and module 3) (**Figures 3B–D**). Module 1 comprised four depleted and eight enriched metabolites in the SV group, such as D-mannose, 2-ketobutyric acid, taurine, and GABA. Nonetheless, they were positively correlated with each other. Module 2 encompassed interactions among two metabolites enriched in VB and three metabolites over-expressed in SV group, while D-galactose in module 3 acted as a link between the other four SV-enriched metabolites and two metabolites that increased in the VB group. Furthermore, KEGG and MetaboAnalyst were comprehensively used to explore the most relevant metabolic pathways. The altered metabolites were enriched or depleted in different metabolomic signaling pathways. With respect to metabolites enriched in SV, 35 metabolic pathways were identified, among which three pathways reached the significance threshold (FDR <0.05), as follows: Butanoate metabolism (including GABA, L-glutamic acid, succinic acid semialdehyde, oxoglutaric acid, and succinic acid), alanine, aspartate, and glutamate biosynthesis (containing succinic acid semialdehyde; L-glutamic acid; GABA; citric acid; succinic acid; oxoglutaric acid), D-glutamine, and D-glutamate metabolism (L-glutamic acid; oxoglutaric acid) (**Figure 4A**). Taken together, these findings show a sub-metabolic pathway from L-glutamic acid to succinic acid within butanoate metabolism and alanine, aspartate, and glutamate biosynthesis pathways and showed that glutamate decarboxylase, 4-aminobutyrate aminotransferase, and succinate-semialdehyde dehydrogenase are required (**Figure 4B**). However, the metabolic pathways constructed based on the metabolites enriched in the healthy group did not achieve the threshold of significance (**Figure 4C**).

### Identification of biomarker panel and correlation with clinical serum parameters

**Figure 5:**
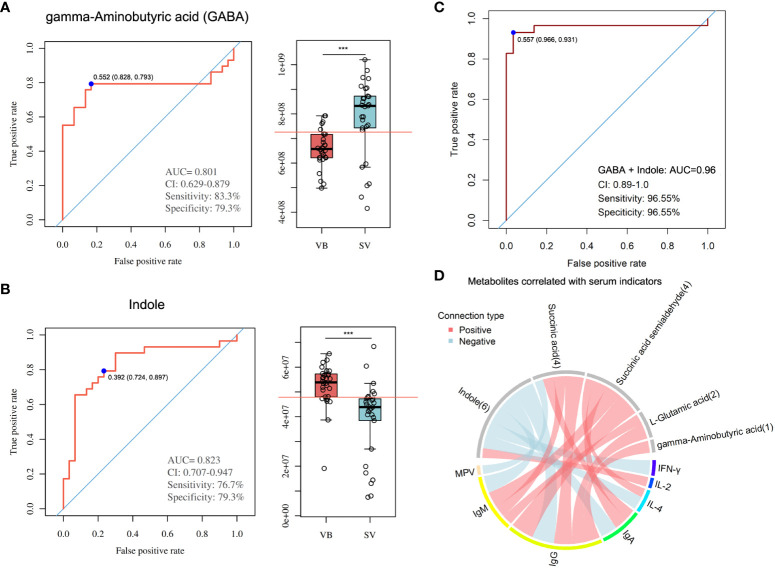
Metabolite markers for pairwise discrimination of the VB and SV groups. **(A-B)** Diagnostic accuracy of the single serum metabolite, GABA, and indole in distinguishing individuals that received two-dose vaccination in the SV group from the VB group. **(C)** The ROC curve with an increased AUC value based on the combination of GABA and indole. **(D)** Associations between serum metabolites and clinical parameters. The associated metabolites are colored gray, and the associated clinical parameters are colored successively. Each line indicates a significant correlation between a metabolite and a clinical parameter, with light red corresponding to a positive association (correlation coefficient ≥ 0.3) and light blue representing a negative association (correlation coefficient ≤-0.03).

To evaluate the reliability of 100 biomarker candidates and define the useful biomarkers, a binary logistic regression analysis and an optimized algorithm of the forward stepwise (Wald) method were employed to construct the best model using 100 differential and potential metabolite biomarkers. For classifying participants in the VB from the SV group, we found that GABA and indole achieved high AUC values of 0.801 and 0.823 (**Figures 5A, B**), respectively. In addition, the combination of GABA and indole increased AUC to a higher degree of 0.96 in distinguishing individuals in the VB group from individuals without vaccination (**Figure 5C**).

Furthermore, we combined the metabolites enriched in butanoate metabolism or alanine, aspartate and glutamate biosynthesis pathways with biomarker metabolic features and performed global correlation analysis with antibodies or biochemical parameters to identify the potential role of metabolites in mediating host immune response and physiology (antibodies such as IgG, IgM, and IgA, and cytokines such as IL-4 and IFN-γ, and blood RT) upon SARS-CoV-2 vaccination. On the whole, we detected that the serum levels of IgM, IgG, IgA, IL-4, and IFN-γ, blood level of MPV showed a negative correlation with indole intensity, while IL-2 was positively correlated with indole. Notably, GABA, L-glutamic acid, succinic acid, and succinic acid semialdehyde, which were found to be enriched in the SV group, was positively associated with IgG. In addition, IgM was significantly associated with succinic acid, succinic acid semialdehyde, and L-glutamic acid. Moreover, the antibody IgA showed a positive correlation with sera succinic acid and succinic acid semialdehyde. Meanwhile, cytokine IL-4 was also positively associated with succinic acid semialdehyde (**Figure 5D**, **Table S4**).

## Discussion

In the process of immune activation, the demand for biomass construction and effector molecule synthesis requires a lot of adjustment of cell metabolism, and numerous small molecules are produced. Compared with the components of classical cell signaling pathways mediated by cytoplasm, membrane-bound, or secreted proteins, small metabolites have great evolutionary potential as communication molecules because they can be synthesized and secreted more quickly with fewer cell resources (33). Recently, a growing number of studies have suggested the increasing acceptance of mass spectrometry-based techniques for interrogating neonatal responses to vaccines (34). However, to date, few published systems vaccinology studies have assessed the systemic metabolic responses of teenagers to immunization with the inactivated SARS-CoV-2 vaccine.

The inactivated SARS-CoV-2 vaccine has been shown to generate rapid and vast antibody responses, for instance, IgG, making it appropriate for emergency usage and critical during the COVID-19 or its variant pandemic (35–37), which is consistent with our study. Peculiarly, we detected a non-significant increase in IFN-γ levels and a reduction in IL-2 levels in the sera of vaccinated individuals. The foregoing results imply that vaccination may not trigger a relatively strong inflammatory response, as is the case with infection, and that such a relatively weak or even downregulation of inflammation may have beneficial effects on the immune reaction of the body in response to the vaccine. Meanwhile, we present the overall characterization of SARS-CoV-2 vaccination-induced changes to the undergraduate student serum metabolome by untargeted metabolomics. In summary, we found that the serum metabolic profiles of vaccinated individuals were significantly different from those before vaccination. A bulk of circulating metabolites are associated with vaccination. Taurine is a naturally occurring sulfur-containing amino acid that can be converted from other amino acids in the liver or obtained from the diet (38). Recently, a growing number of studies have found that taurine plays a critical role in regulating immune system health and antioxidation and has been reported to have anti-inflammatory effects by suppressing cytokine production (39–41). Intriguingly, taurine was remarkably upregulated in vaccinated individuals in our results. The strong and positive relationship between taurine and an immunized antibody level reinforces the suggestion that vaccination can establish a reciprocal regulatory relationship between taurine and neutralizing antibodies. The observations in this study are somewhat in concert with the results of previous research in relation to BCG-induced protective effects as with taurine (42). However, this is the first study implicating a potential role for taurine in response to SARS-CoV-2 inactivated vaccine-induced immunity, but the mechanism of action remains elusive. It is noteworthy that taurine was first identified to respond to the immune response after receiving the SARS-CoV-2 inactivated vaccine; nonetheless, the underlying mechanism of action is not clarified.

In our study, SARS-CoV-2 vaccine-induced changes in metabolite pathways correlated with the blood antibody and cytokine responses. It has been well documented that the tricarboxylic acid cycle and amino acid metabolism exert an essential influence in the modulation of the immune response to vaccines (43–45). In addition to the TCA cycle and amino acid metabolism pathways that are already known to influence host adaptive immunity, one of the most enriched metabolic pathways which was significantly associated with immune responses upon SARS-CoV-2 vaccination is glutamate metabolism. GABA is a non-protein amino acid comprising four carbon atoms that can be produced by plants, animals, and microorganisms (46, 47). In mammals, GABA has been well studied, primarily as a neurotransmitter (48, 49). Meanwhile, the receptor of GABA (GABA-Rs) plays a pivotal role in the neurotransmission process in the central system. Nevertheless, a recent report revealed that GABA-Rs can be expressed in the immune and epithelial cells as well and showed that GABA treatment can ameliorate inflammation and reduce viral load in the lungs (50). Simultaneously, our findings identified that GABA and its predecessor metabolite (L-glutamic acid) were significantly upregulated in vaccinated individuals, and GABA also has a significant positive correlation with the serum level of IgG. Specifically, GABA is known to be transformed form L-glutamic acid by glutamate decarboxylase (GAD). Numerous studies have shown that probiotics, such as *Lactobacillus*, possess CAD genes in their genome and are effective in producing large amounts of GABA (51–53). Notably, a very recent study revealed that *Lactobacillus plantarum* GUANKE (LPG) could foster SARS-CoV-2 specific immune reactions in vaccinated mice by potentiating interferon signaling, inhibiting apoptosis and inflammatory pathways. The administration of LPG to mice after the inoculation of the SARS-CoV-2 vaccine can boost and maintain the production of SARS-CoV-2-neutralizing antibodies in their bodies (54). The results described above indicate that a direct supplementation with GABA, oral administration of probiotics capable of generating GABA and stimulating antibody production, or targeting GABA-Rs could strengthen the neutralizing antibody production in the human body in response to SARS-CoV-2 vaccination. Yet, the precise regulatory mechanisms should be investigated in greater detail. In addition to GABA, the other three metabolites (L-glutamic acid, succinic acid semialdehyde, and succinic acid) enriched in the glutamate metabolism pathway were remarkably elevated in vaccinated individuals, and all of them were significantly positively correlated with the levels of either IgM, IgG, or IgA. This further led us to speculate that these metabolites may be closely related to the immune response of the organism after immunization.

In this investigation, the combination of two metabolite biomarkers we found, GABA and indole, was able to determine whether an individual had been inoculated with the SARS-CoV-2 vaccine or not with a 95.6% accuracy based on AUC value. Therefore, in the future, if we want to identify whether a population has been vaccinated with the SARS-CoV-2 vaccine on a large scale, our tentative guess is that we can base on the detected concentrations of GABA and indole in the serum of individuals. Despite the existence of indole and GABA as both metabolite markers to distinguish individuals from vaccinated individuals, a significantly negative relationship pattern was detected between indole and all neutralizing antibodies in the serum, which is contrary to the results for GABA. Indole is formed by the bacterial metabolism of tryptophan, and currently the majority of research has reported its relatively potent anti-inflammatory immune effects (55, 56). In general, the negative correlation between indole and antibodies and the significant reduction of indole in the serum of vaccinated individuals only suggest that immunizations prevent indole from impeding the production of neutralizing antibodies in the body by reducing indole levels.

Although we were successful in recruiting 30 college student volunteers to comprise the research cohort in this study and collecting blood or serum samples before SARS-CoV-2 vaccination and after receiving two doses of the vaccine. However, the cohort was still relatively small in size to obtain the best reliability. In addition, to better confirm our findings, metabolic analysis of serum samples from larger COVID-19-vaccinated cohorts and participants of various ages may be warranted.

Taken together, our metabolomic data presented in this study provide an overall view of circulating metabolite alteration from volunteers who received two doses of the inactivated SARS-CoV-2 vaccine as compared to that before vaccination. We identify four metabolites enriched in either butanoate metabolism or the alanine, aspartate, and glutamate metabolism pathways as the mainly responding small molecules upon vaccination. Correlation analysis further indicates that L-glutamic acid, GABA, succinic acid semialdehyde, and succinic acid could be used as a small molecules for potential targeted intervention to augment the immune response after SARS-CoV-2-based immunization or that of its variant.

## Data availability statement

The datasets presented in this study can be found in online repositories. The names of the repository/repositories and accession number(s) can be found below: https://www.ebi.ac.uk/metabolights/index, accession ID: MTBLS4995.

## Ethics statement

The studies involving human participants were reviewed and approved by the ethics committee of Anhui Medical University Biomedical Ethics Committee (Approval No. 2021H021). The patients/participants provided their written informed consent to participate in this study.

## Author contributions

SH and WJ conceived and designed the experiments and revised the manuscript; MHe, YH, and YW performed the experiments, analyzed the data, and wrote the manuscript; JL, MHa, YX, NZ, HG, HQ, and LC collected the samples and performed the experiments. All authors read and approved the final manuscript.

## Funding

This work was supported by Grants for the Natural Science Foundation of China (No.81974306), Major Project of Natural Science Research of Anhui Education Department (No. KJ2019ZD23), the Project of Quality Engineering of Anhui Province (No. 2019zyrc017, 2020jyxm0864, 2021jcxkpy013), General Program of Scientific Research in Higher Education Institutions of Anhui Province (ZR2019B04), Basic and Clinical Collaboration Enhancement Program Foundation of Anhui Medical University (2020xkjT023), and Grants for Scientific Research of BSKY (XJ201922) from Anhui Medical University.

## Conflict of interest

The authors declare that the research was conducted in the absence of any commercial or financial relationships that could be construed as a potential conflict of interest.

## Publisher’s note

All claims expressed in this article are solely those of the authors and do not necessarily represent those of their affiliated organizations, or those of the publisher, the editors and the reviewers. Any product that may be evaluated in this article, or claim that may be made by its manufacturer, is not guaranteed or endorsed by the publisher.
