# Supplementary material for: Metabolomics-based investigation of SARS-CoV-2 vaccination (Sinovac) reveals an immune-dependent metabolite biomarker
Source: Front Immunol. 2022 Sep 28;13:954801. doi: 10.3389/fimmu.2022.954801 (PMC9554639; doi:10.3389/fimmu.2022.954801)
Supplement: Supplementary file 1 [file Presentation_1.pdf]

Metabolomics-based investigation of SARS-CoV-2 vaccination (Sinovac) reveals immune dependent metabolite biomarker

Maozhang He[†1], Yixuan Huang[†2], Yun Wang[†4], Ling-Ji Liu[1], Maozhen Han[3], Yixuan Xiao[1], Na Zhang[3], Hongya Gui[1], Huan Qiu[5], Liqing Cao[3], Weihua Jia[*3], Shenghai Huang[*1,3]

[*]To whom correspondence should be addressed

[1]Department of Microbiology, The Key Laboratory of Microbiology and Parasitology of Anhui Province, The Key Laboratory of Zoonoses of High Institutions in Anhui, School of Basic Medical Sciences, Anhui Medical University, No. 81 Meishan Road, Hefei 230022, P.R. China.

[2]Department of Clinical Medicine, The First School of Clinical Medicine, Anhui Medical University, No. 81 Meishan Road, Hefei 230022, P.R. China.

[3]School of Life Sciences, Anhui Medical University, No. 81 Meishan Road, Hefei 230022, P.R. China.

[4]Anhui No.2 Provincial People's Hospital, Hefei, 230000, Anhui Province, P.R. China.

[5]School of Nursing, Anhui Medical University, Hefei, 230000, Anhui Province, P.R. China.

[†]Maozhang He, Yixuan Huang, and Yun Wang contributed equally to this work.

[*]Correspondence authors: Shenghai Huang, E-mail: huangshh68@aliyun.com. Weihua Jia jwhua29@163.com.

Phone: 0086-551-65172100; Fax: 0086-551-65172100

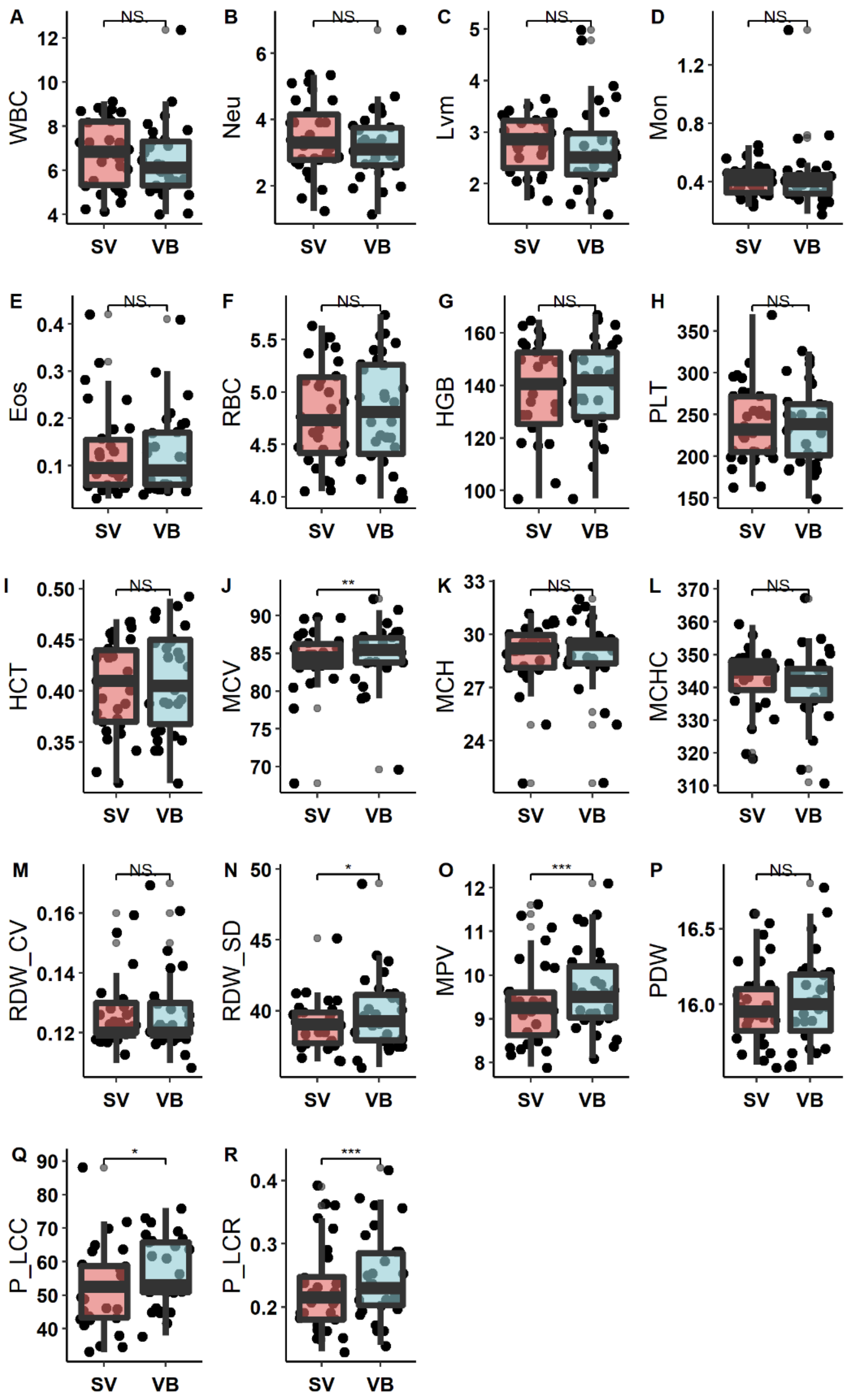

Figure S1. Eighteen blood routine indicators of participants in the VB group and individuals in the SV group.

Significance indicated by the asterisks (unpaired two-sided Welch's t test. p value: *, < 0.05; **, < 0.01; ***, < 0.001. While NS indicates non-significant difference.)

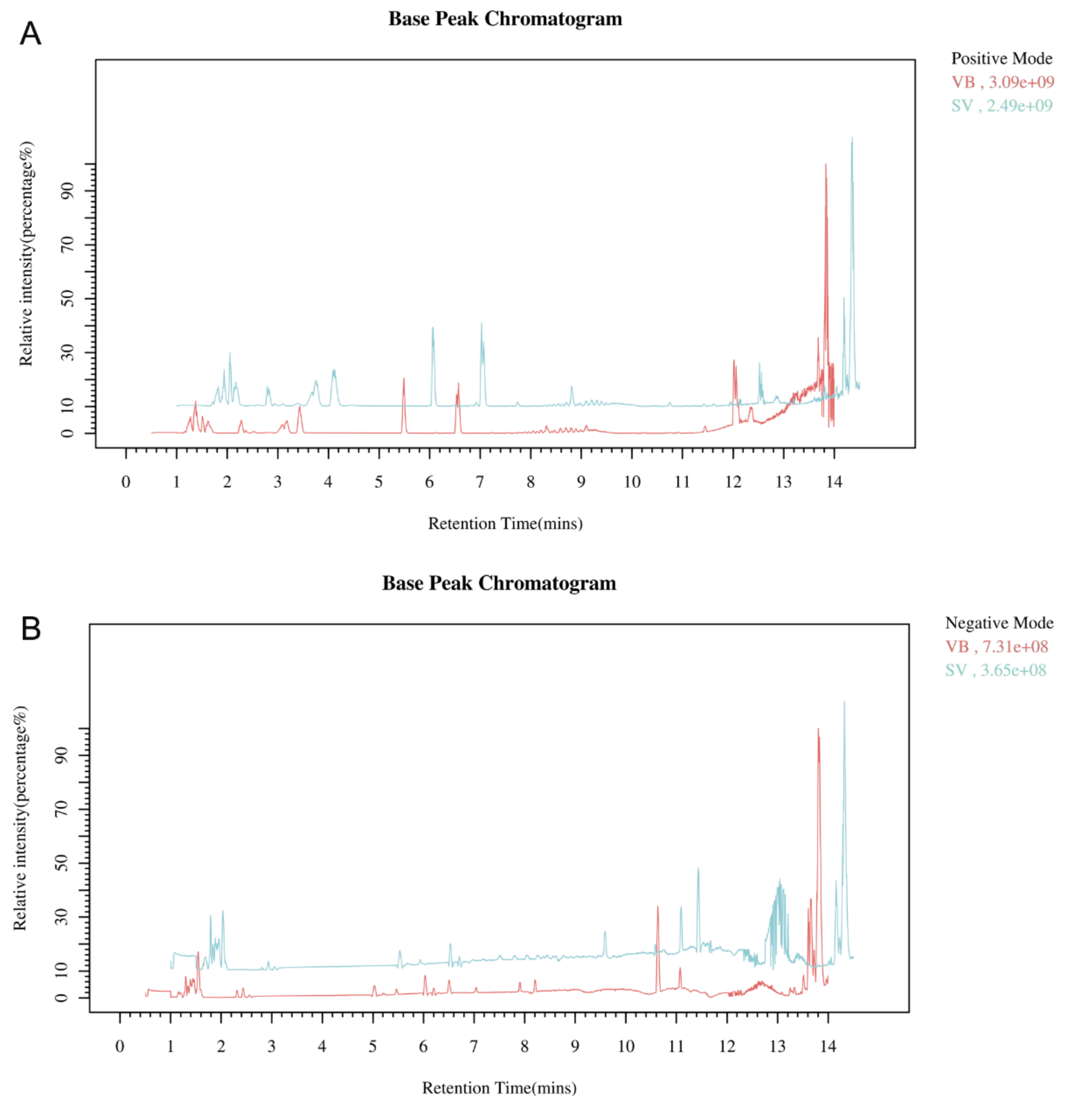

Figure S2. Typical Base Peak Chromatogram of the ESI+ (A) and ESI- (B) modes.

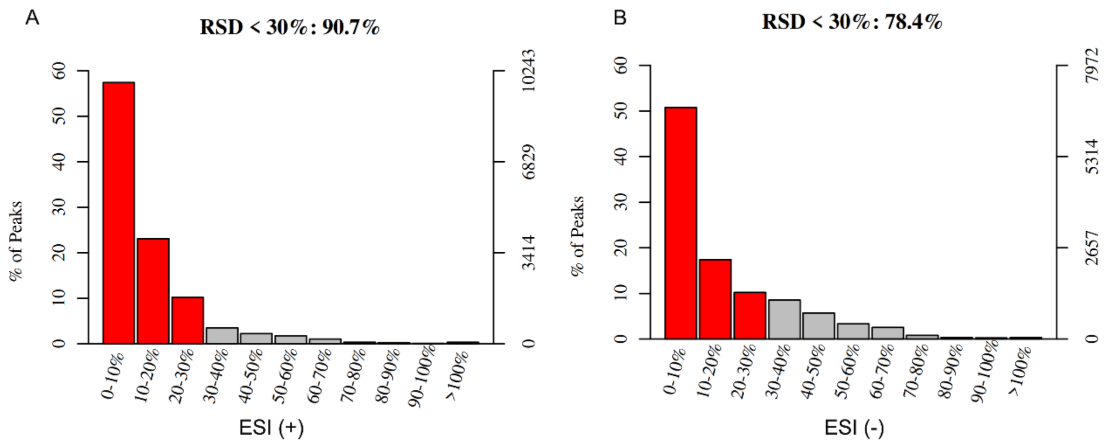

Figure S3. CV distribution of peaks in combinational dataset of ESI+ and ESI- modes. The columns represent the number of peaks and accumulative percentage of peaks in corresponding CV interval, respectively. After removing the missing values

using the 80% rule, and signal correction using the local linear regression (LoReg) method, 90.7% and 78.4% peaks had coefficients of variation (CV) below 30% in ESI+ (A) and ESI- (B) modes, respectively.

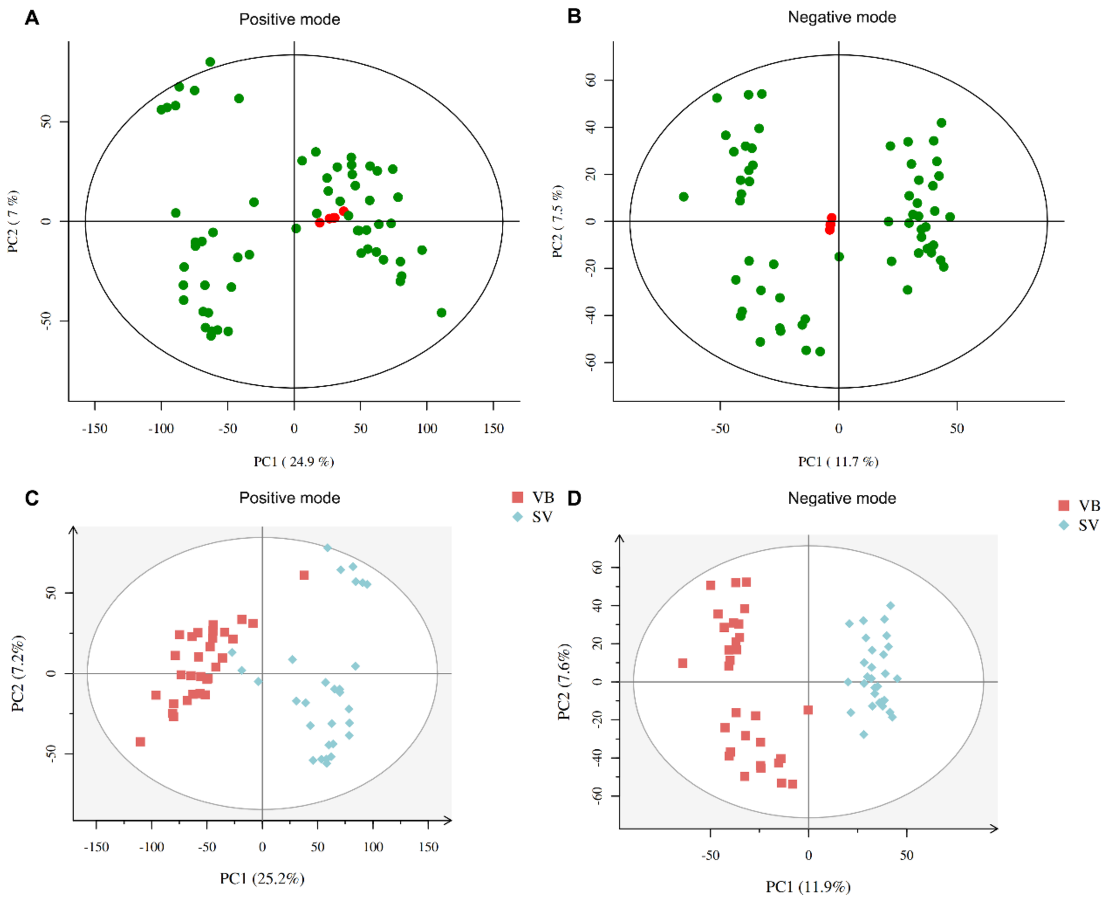

Figure S4. Score plots of principal component analysis based on the ESI+ and ESI− modes. (A-B) Red solid circle represented the QC samples. (C-D) Colors and shapes display the subjects from different groups.

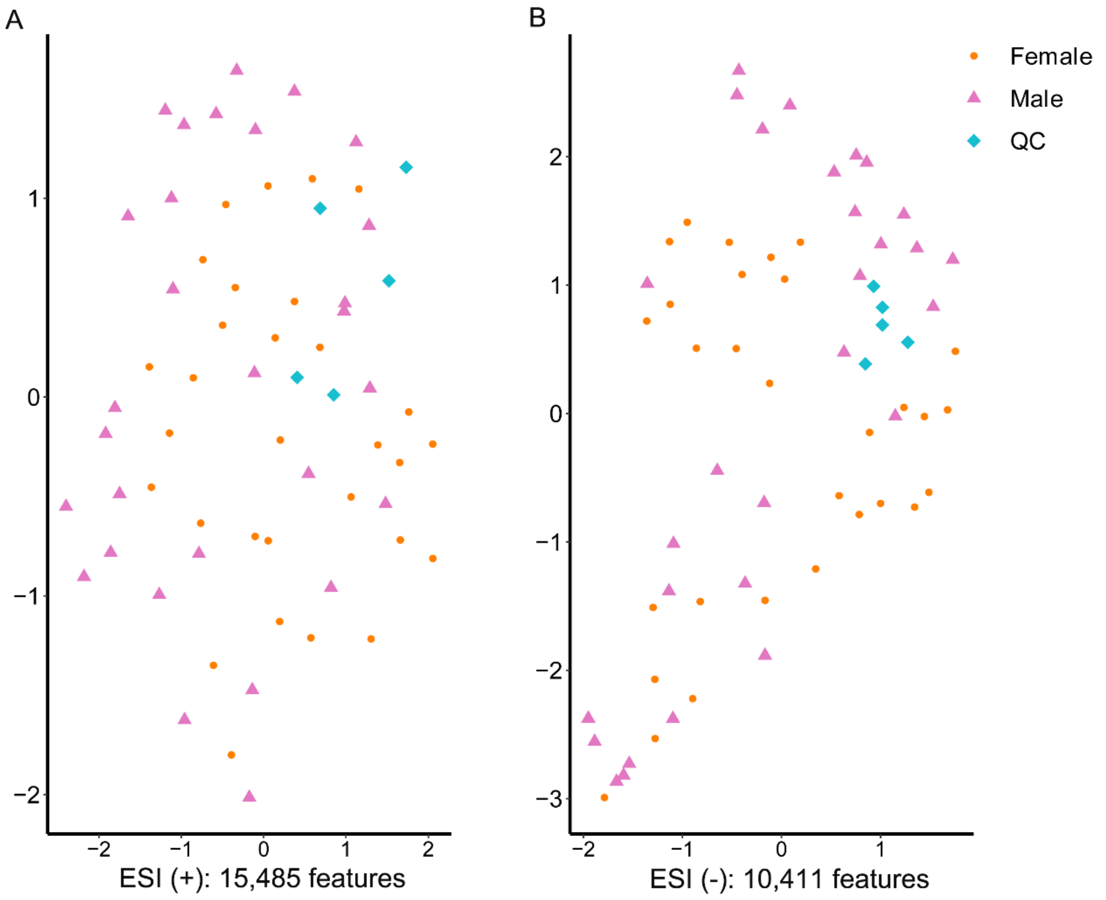

Figure S5. (A) Uniform Manifold Approximation and Projection (UMAP) of sera samples using 15,485 metabolic features in positive mode. (B) UMAP of sera samples using 10,411 metabolites in negative mode.

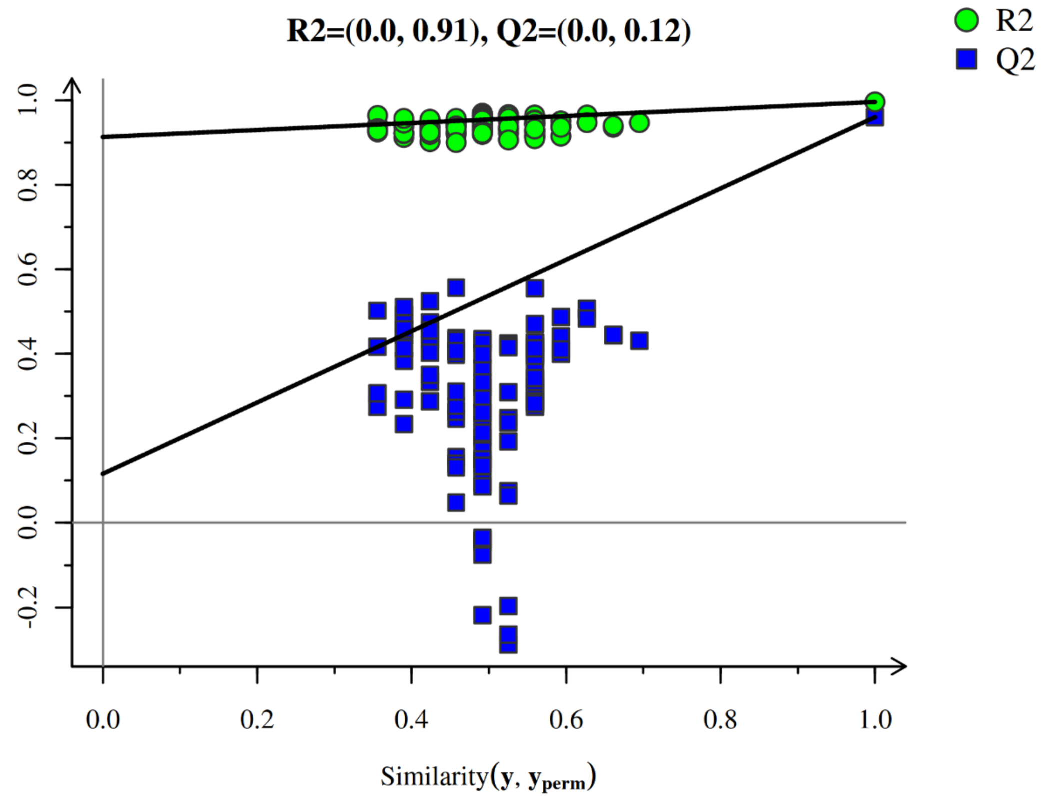

Figure S6. Validated plot of response permutation testing. The Y-axis designates R2Y (triangles) and Q2Y (squares) for PLSDA model. The X-axis represents the correlation coefficient between original and permuted response data.

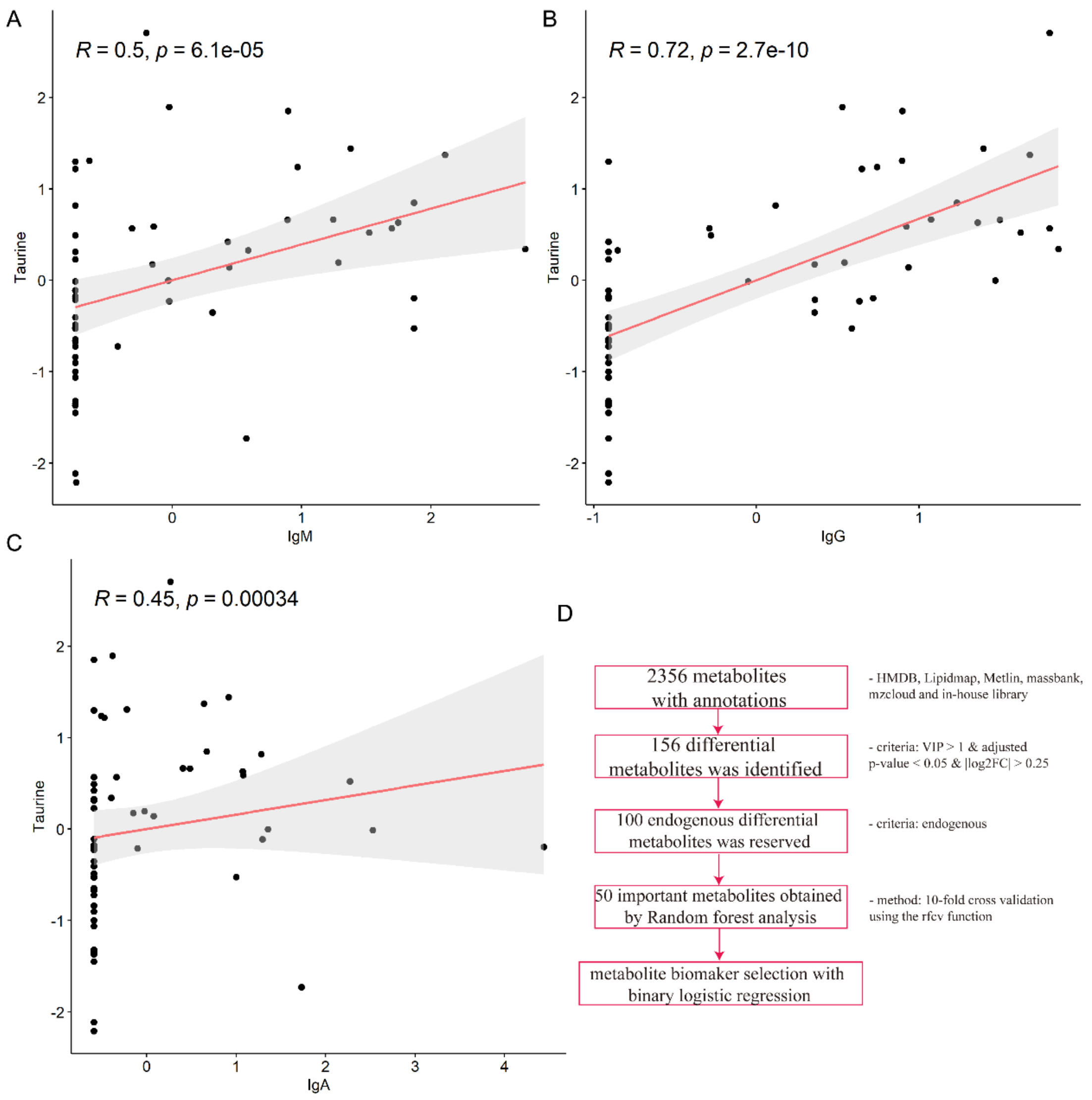

Figure S7. Scatter plot in related to the correlation between serum taurine and neutralizing antibody: (A-C) IgM, IgG and IgA. (D) A flow chart showing the procedure of metabolite biomarkers selection.

Table S1. Overview of the eighteen blood routine indicators in VB and SV groups.

Table S2. Detailed annotation information of metabolite features and the intensity across individuals.

Table S3. Correlation relationships among the metabolites that expressed differentially between SV and VB groups.

Table S4. The correlation coefficients and FDR value in regarding to the correlation between serum metabolites and distinct antibodies, cytokines and blood routine indicators.
